# Free iliac crest grafting technology for the management of critical-sized tibial bone defect

**DOI:** 10.1186/s12891-024-07335-y

**Published:** 2024-03-07

**Authors:** Xiaoqiang Cheng, Yilun Yao, Kang Liu, Lei Wu, Wengbo Yang

**Affiliations:** Department of Orthopaedics, Nanjing First Hospital, Nanjing Medical University, Nanjing, 210000 China

**Keywords:** Critical-sized tibial bone defect, Iliac crest bone graft, Operation skill, Bone resorption

## Abstract

**Objective:**

To introduce the method and experience of treating critical-sized tibial bone defect by taking large iliac crest bone graft.

**Methods:**

From January 2020 to January 2022, iliac crest bone grafting was performed in 20 patients (10 men and 10 women) with critical-sized tibial bone defect. The mean length of bone defect was 13.59 ± 3.41. Bilateral iliac crest grafts were harvested, including the inner and outer plates of the iliac crest and iliac spine. The cortical bone screw was used to integrate two iliac bone blocks into one complex. Locking plate was used to fix the graft-host complex, supplemented with reconstruction plate to increase stability when necessary. Bone healing was evaluated by cortical bone fusion on radiographs at follow-up, iliac pain was assessed by VAS score, and lower limb function was assessed by ODI score. Complications were also taken into consideration.

**Results:**

The average follow-up time was 27.4 ± 5.6 (Range 24–33 months), the mean VAS score was 8.8 ± 1.9, the mean ODI score was 11.1 ± 1.8, and the number of cortical bone fusion in the bone graft area was 3.5 ± 0.5. Satisfactory fusion was obtained in all cases of iliac bone transplant-host site. No nonunion, shift or fracture was found in all cases. No infection and bone resorption were observed that need secondary surgery. One patient had dorsiflexion weakness of the great toe. Hypoesthesia of the dorsal foot was observed in 2 patients. Ankle stiffness and edema occurred in 3 patients. Complications were significantly improved by physical therapy and rehabilitation training.

**Conclusion:**

For the cases of critical-sized tibial bone defect, the treatment methods are various. In this paper, we have obtained satisfactory results by using large iliac bone graft to treat bone defect. This approach can not only restore the integrity of the tibia, but also obtain good stability with internal fixation, and operation skills are more acceptable for surgeons. Therefore, it provides an alternative surgical method for clinicians.

## Background

The treatment of critical-sized tibial bone defect (CSBD) caused by high-energy injury is still a difficult problem. The strong desire of patients for lower limbs preservation and the requirement of limb function causes a challenge to the surgeons. Treatment approach depends on the surgical technique and experience of the surgeons, causing there is no large clinical trials or treatment guidelines raising a treatment standard.

Existing clinical strategies for CSBD, including Ilizarov technique, free vascularized fibula graft (VFG) and Masquelet technique [1]. VFG has been widely used in clinical practice for its abundant blood supply, which can significantly shorten the bone healing time [2]. VFG can be applied to almost all types of bone defects with a length of 26-28 cm [3]. Compared with other bone grafts, VFG has stronger biomechanical strength, better osteogenic induction and osteogenic ability. In addition, the diameter of the fibula is more similar to that of the tibial pulp cavity, contributing higher fibula-tibia fusion rate. However, the VFG has its limitations. CSBD caused by high-energy injuries are often accompanied by fibula fractures and vascular injury, therefore VFG in contralateral limb can be a potential choice, causing inevitable trauma in healthy limb and potential incidence of infection, common peroneal nerve injury, ankle instability and valgus deformity [4]. Recent research reveals the incidence of complications in VFG surgery was 37–80% [5, 6], including occurrence of nonunion, fracture and infection (top 3 incidence) in the transplant and donor area [7].

Ilizarov technique has been widely used in the clinical treatment of CSBD with mechanism of distraction osteogenesis [8, 9]. With the continuous and slow extension stress on the osteotomy location, the gap in the osteotomy area that is slowly stretched is filled with new callus tissue. Patients underwent Ilizarov surgery can rehabilitate lower limb function at an early stage due to the good stability provided by external fixation, which greatly reduces the incidence of muscle atrophy, thrombosis and other complications [10]. However, Ilizarov technique has its limitations, including its surgical procedures and postoperative care [11], long treatment cycles, infection and loosening of the pin tract. In addition, external fixators limit the functional rehabilitation of the knee and ankle joints, resulting in joint stiffness that can cause trouble for patients later in life [12].

Iliac crest bone grafting (ICBG) is widely used for the treatment of limited bone defects [13, 14]. We tried to take free avascular large segment iliac crest bone blocks for the treatment of CSBD and achieved a satisfactory result. Due to the biological characteristics of the iliac crest as a lamellar bone, the inner plate, outer plate, iliac spine (3 bone cortices) and cancellous bone located deep in iliac crest constitute the free iliac crest blocks. The rigidity of cortical bone and its resistance to degradation ensure the physical support in the graft area. Cancellous bone is rich in osteoprogenitor cells and osteogenesis induced factor to ensure the bone healing rate in the bone graft area [15]. The patient can proceed the early rehabilitation without sacrificing the function of the knee and ankle joint with internal fixation. Simple surgical methods are more accessible to the surgeon.

## Materials and methods

### Patient

A retrospective study (*n* = 20; 10 men and 10 women) was conducted on patients with CSBD in the middle tibia underwent free ICBG from January 2020 to January 2022 in our hospital. We defined CSBD with a defect length (> 5 cm) [16] that could not be effectively treated with simple bone grafting accompanied with internal fixation. The mean length of tibial bone defect was 13.59 ± 3.41 cm (Range 10.5-17.0 cm). The treatment approach of CSBD was free avascular iliac crest bone grafting fixed with locking compression plate. All cases were followed up for more than two years, and the mean follow-up time was 27.4 ± 5.6 months (Range 24–33 months).

### Inclusion and exclusion criteria

All skeletally mature patients over 18 years of age with primary non-pathological tibial fractures were included in the study. The tibial defect was caused by primary injury. Patients were excluded from the study if they had any drainage fistula or infection, as determined by erythrocyte sedimentation rate, serum C-reactive protein, and white blood cell levels, or if there was radiological evidence of septic bone nonunion and the bacterial culture sample was positive for deep infection. Cases with low adherence and less than 24 months of follow-up were excluded.

### Surgical procedure

Soft tissue defects were observed in all cases. Flap transplantation was used to solve the problem. Secondary surgery for tibial defect was performed after flap transplantation. Details of flap transplantation was omitted in the paper causes it is not the key point.

After disinfection, the incision should be extended appropriately according to the wound caused by the original trauma, avoiding vascular injury. The skin and soft tissue are pulled to expose the proximal and distal tibial fractures, removing the scar tissue, refreshing the tibial fracture surface, recanalizing tibial medullary cavity. The proximal and distal tibia were grooved for subsequent manipulation (slot length:1-2 cm, width:2 cm). A curved incision was performed to expose the medial malleolus, retrogradely inserting the medial tibial anatomical plate. The lower limb reduction was reached by continuous traction in consideration of tibia length and lower limb alignment, screwing the screws with minimally invasive percutaneous plate osteosynthesis (MIPPO) technology. C-arm fluoroscopy was used to confirm the fixation of bone graft-host area.

**Fig. 1:**
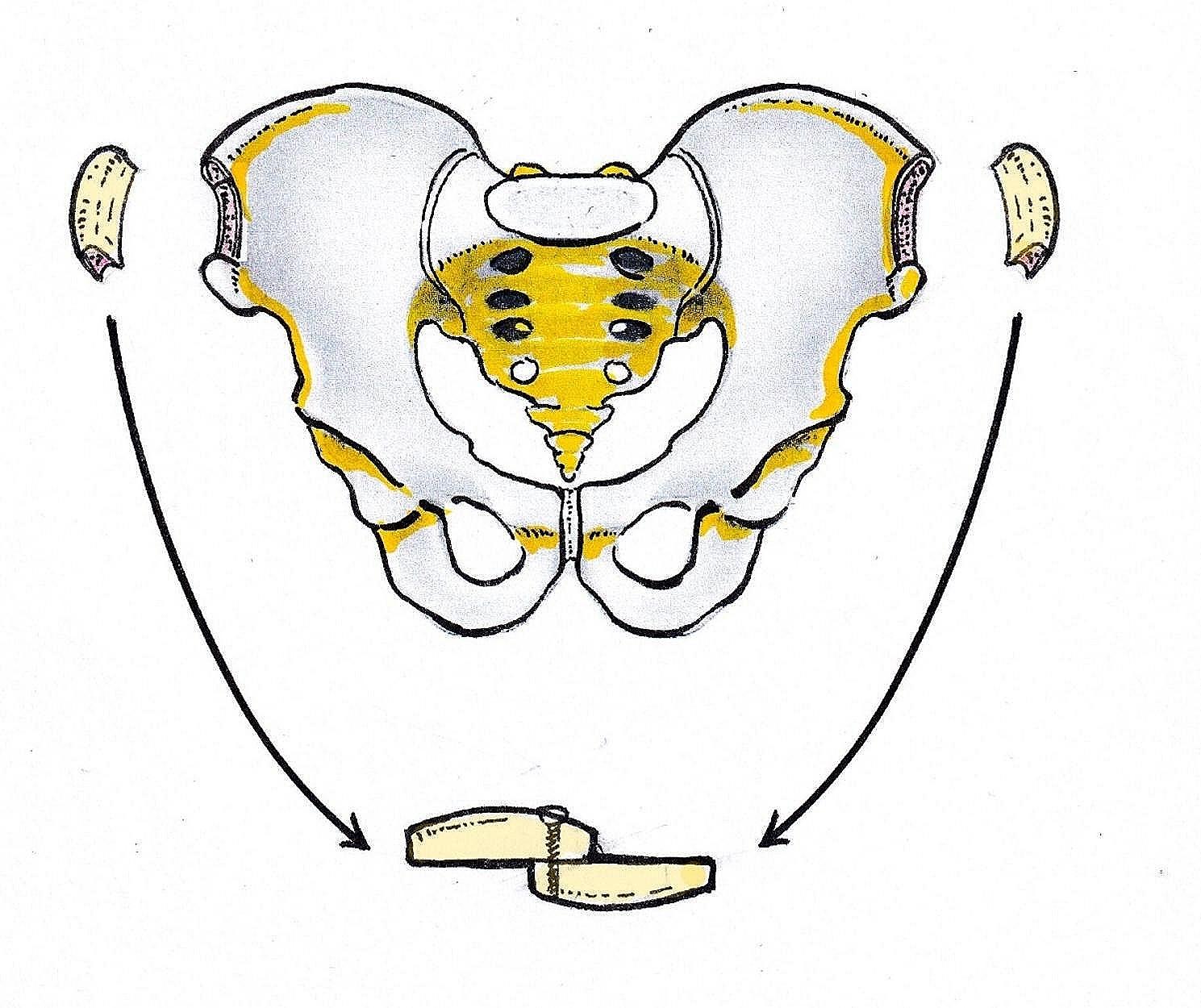
Two iliac crest blocks harvested from bilateral ilium. Two blocks were fixed by a 3.5 mm cortical screw to shape a longer strip

**Fig. 2:**
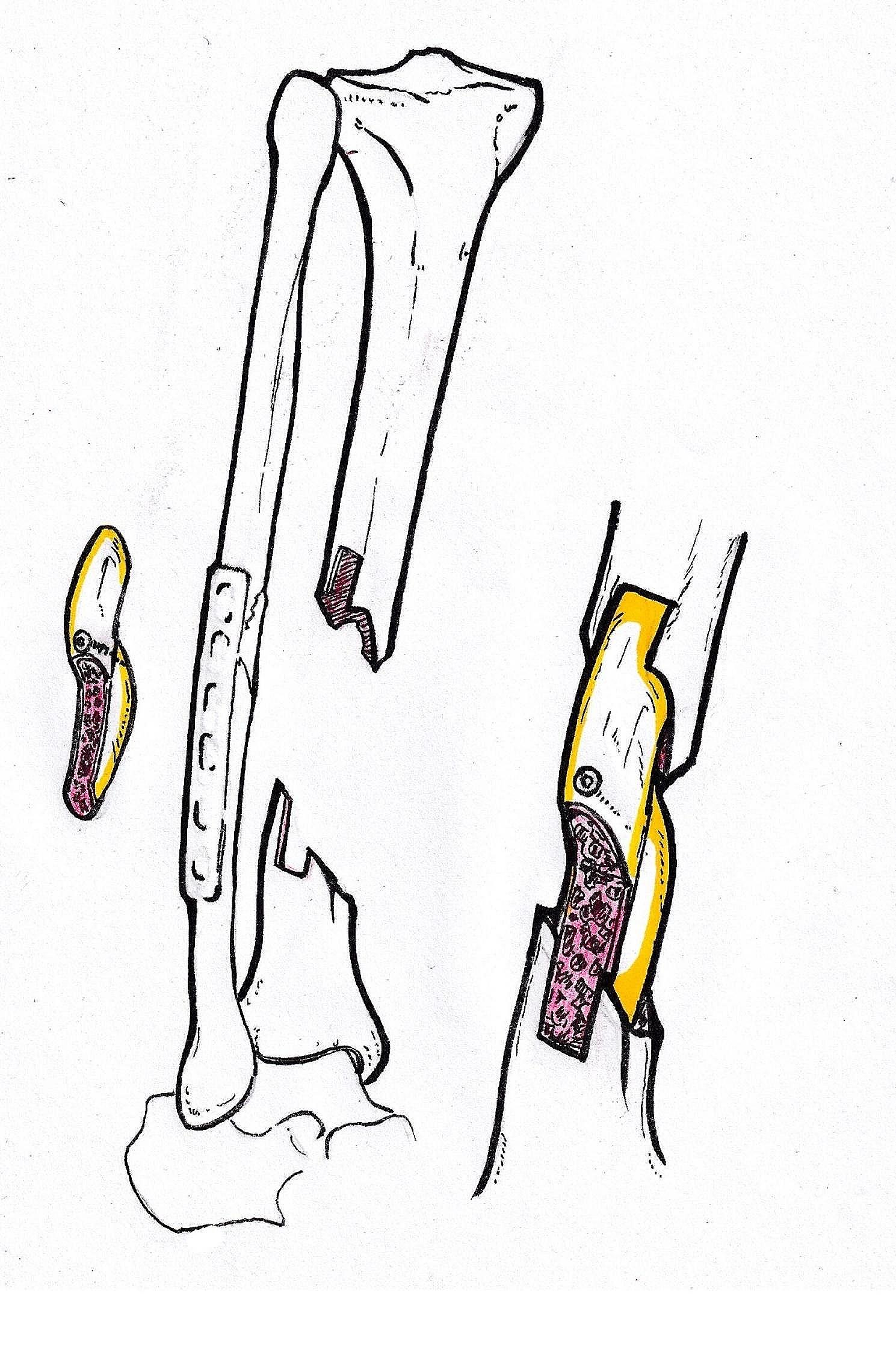
Repair of the bone defect. The shaped iliac crest block was stuck in the prepared slot in tibia

**Fig. 3:**
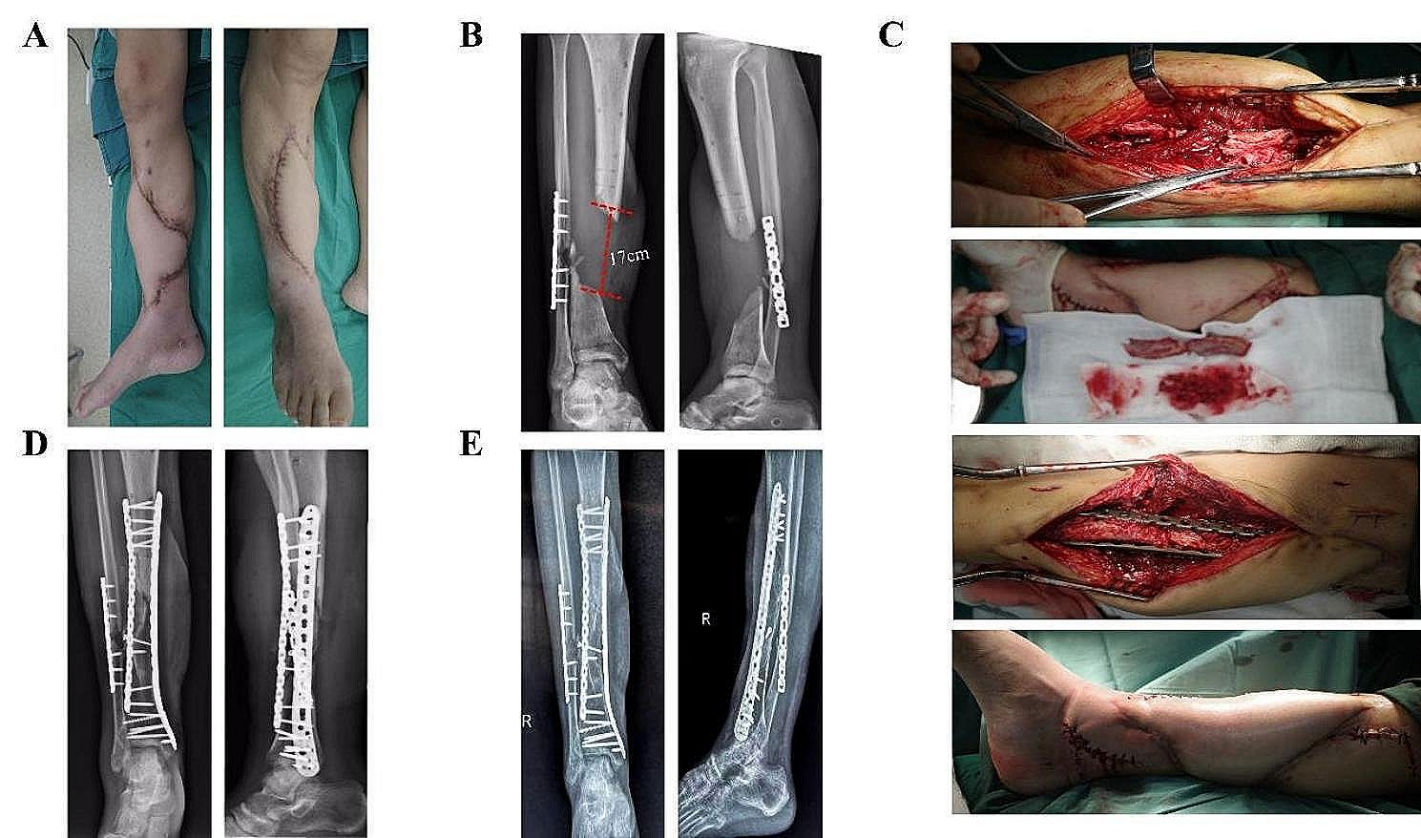
(**A**) Repair of lower limb wound defect with skin flap. (**B**) X -ray examination shows bone defect was 17 cm before operation. (**C**) Exposure of the tibial defect, iliac crest shape and graft, internal fixation with plate. (**D**) X-ray examination after operation before discharge. (**E**) X -ray examination at 12-month follow-up

**Fig. 4:**
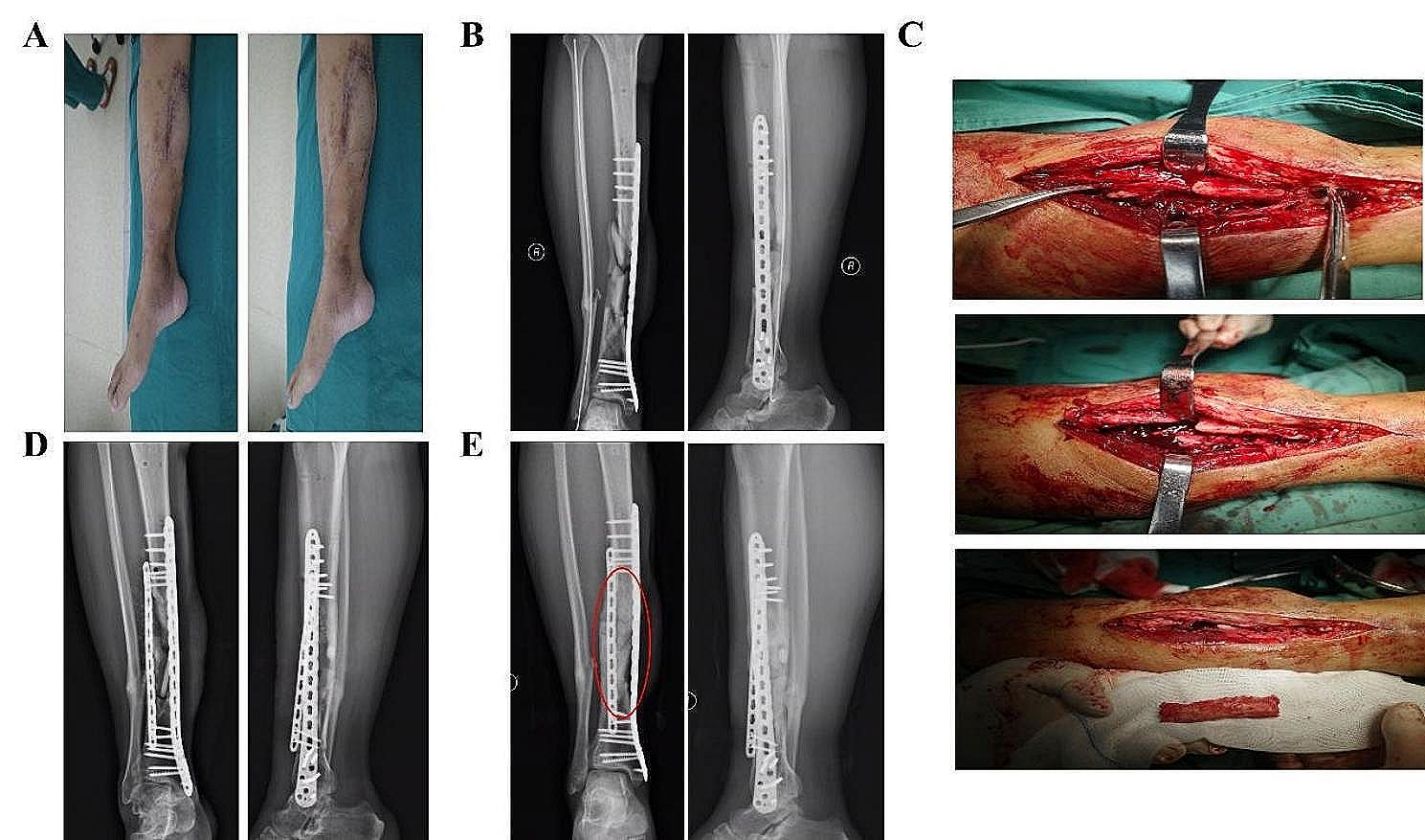
(**A**) Repair of lower limb soft tissue defect with Skin flap transplantation. (**B**) X-ray examination before surgery of bone reconstruction. (**C**) Exposure of bone defect and iliac crest harvesting. (**D**) X-ray examination after surgery. (**E**) X -ray examination at 6-month follow-up

**Fig. 5:**
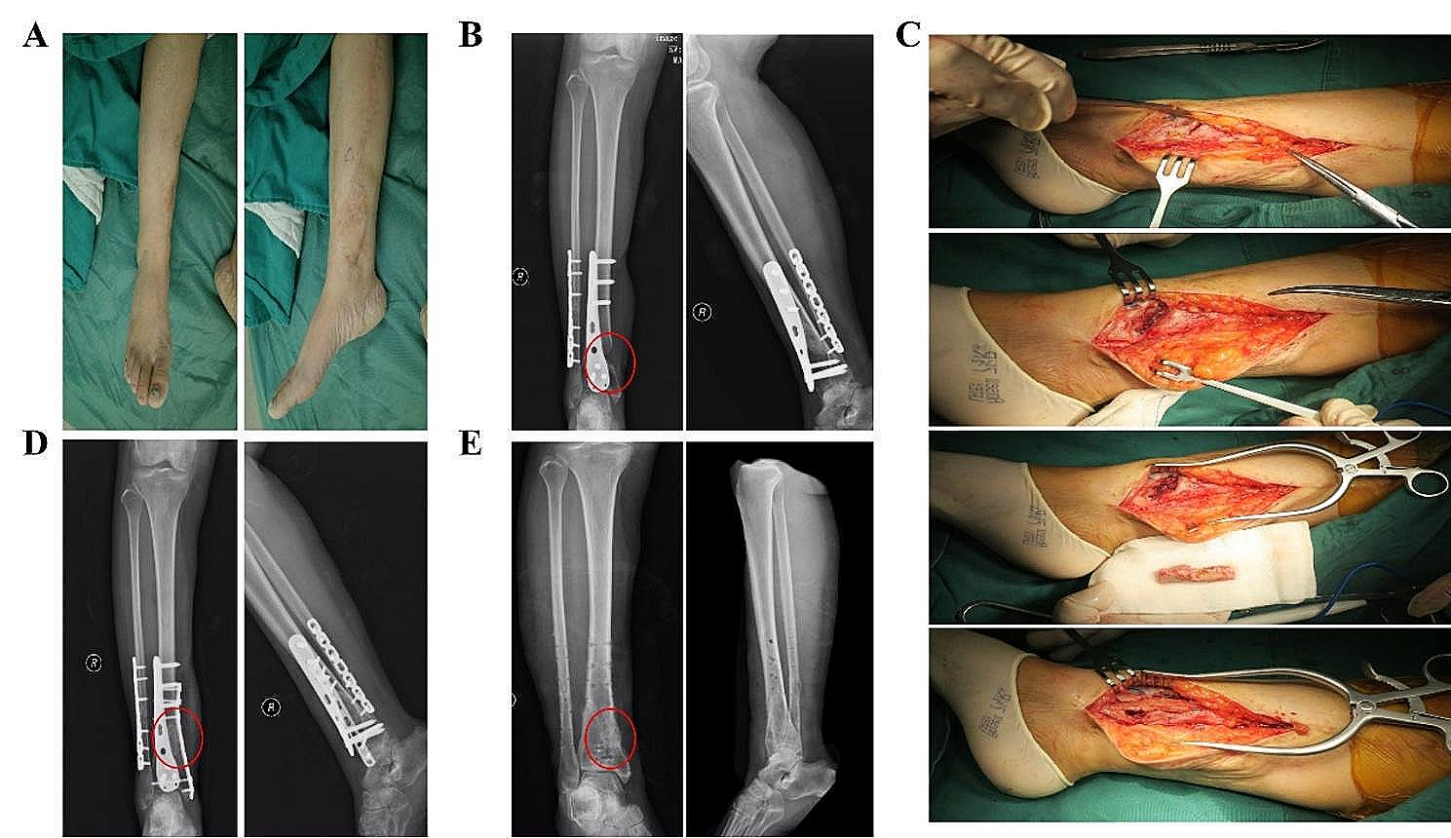
(**A**) Soft tissue defect was successfully repaired. (**B**) X-ray examination before bone reconstruction. (**C**) Exposure the tibial defect and bone grafting procedure. (**D**) X-ray examination at 6-month follow-up. (**E**) Internal fixation was removed 12 months after surgery

An incision was made 3 cm posterior to bilateral anterior superior iliac spine, Osteotomy was performed after removing the soft tissue attached to the iliac crest. A gauze was used for tamponade to stop bleeding. Make sure there is no active bleeding before closing the incision. As is shown in Fig. 1, iliac bone grafted block (Width:2 cm) consists of 4 parts: inner cortical plate, outer cortical plate, iliac spine and cancellous bone. The length of the iliac bone graft (up to 10 cm) depends on the size of tibial bone defect (up to 17 cm). Removing the cortex of the iliac crest that will be inserted into the tibial slot (mentioned above). As is shown in Fig. 2, two iliac crest grafted bone were fixed with a 3.5 mm cortical bone screw to shape a longer bone strip (up to 17 cm), inserting the prepared tibial bone slot. The remaining bone defects were filled with cancellous bone, allogeneic bone and bone morphogenetic protein-2 (BMP-2). The reconstruction plate was fixed on the lateral side of the tibia grafted bone area when necessary, inserting screws to increase stability. The C-arm machine fluoroscopy was used to confirm the length of the tibia and the reestablishment of the lower limb alignment. In Fig. 3, we present the treatment of a typical case with the defect of 17 cm. In Figs. 4 and 5, we present cases of smaller but more common tibial bone defects.

### Postoperative management

All patients underwent joint function exercise and muscle strength training after surgery. Mannitol and flurbiprofen ester were used to relieve edema and pain in the affected limbs. Cefazolin sodium was used to prevent infection. Low molecular heparin was used to prevent deep vein thrombosis. Erythrocyte sedimentation rate, procalcitonin, C-reactive protein and interleukin-1,6 were used to monitor the inflammation and infection. No obvious abnormalities in laboratory indicators and no exudate in the affected limb wound were observed before discharge. After discharge, a follow-up of not less than 24 months was conducted through the outpatient clinic, telephone and wechat platform.

### Outcome evaluation

Bone graft healing was assessed by X-ray at each follow-up visit. Postoperative limb function was evaluated by Oswestry Disability Index (ODI), and postoperative pain in iliac region was evaluated by Visual Analogue Scale (VAS) score. Bone fusion and function was measured by Paley grading [17], which was divided into 4 grades: excellent, good, general and poor. Excellent: fracture healing, no infection, local deformity angle less than 7°, limb length discrepancy less than 2.5 cm, no obvious lameness, slight joint stiffness, knee extension angle or ankle dorsiflexion Angle reduced less than 15° compared with the operation, no reflex dystrophy. Good: fracture healing, no infection, local deformity angle less than 7°, obvious claudication, reflex sympathetic dystrophy, obvious pain. General: fracture healing, no infection, able to complete normal daily activities, obvious claudication and reflex sympathetic dystrophy. Poor: fracture nonunion, infection, local deformity angle greater than 7°, significant claudication, reflex sympathetic dystrophy, significant pain.

On the basis of anteroposterior and lateral radiographs of the lower extremity, successful bone fusion was defined as fusion of the graft with 3–4 cortices of the graft-host complex, and the rest were considered as nonunion. If the cortex was occluded by the plate on the X-ray, the oblique radiographs of the target site were used for evaluation.

### Statistical analysis

All data were presented as mean ± standard deviation (SD), All statistics were performed using SPSS 22.0 software.

## Result

The age, sex, length of bone defect, and follow-up time of participants in the study are summarized in Table 1. All cases were open fractures with soft tissue defects, 3 cases were combined with femoral shaft fracture, 1 case with popliteal artery injury, 2 cases with chest trauma, and 2 cases with craniocerebral injury. All bone reconstruction operations were performed after treatment of the patient’s combined injury. The average length of bone defect was 13.59 ± 3.41 (Range from10.5-17.0 cm). The pain in the iliac bone harvesting area was evaluated by VAS score, which was 77.2 ± 4.4 on the first day after surgery, significantly reduced to 29.2 ± 2.2 at the 3-week follow-up, and 8.8 ± 1.9 at the 24-month follow-up. The pain in the iliac bone harvesting area was acceptable. Lower extremity function was evaluated by ODI score. The ODI of the patient on the first day after surgery was 84.7 ± 6.6, indicating the lower extremity was nonfunctional. At the six-month follow-up, the ODI score was reduced to 32.5 ± 3.1, indicating significant improvement in lower extremity function compared with the three-month follow-up. At the two-year follow-up, the patient’s ODI decreased to 11.1 ± 1.8, and the recovery of lower limb function was satisfactory. VAS and ODI scores for each follow-up time were summarized in Table 2. During the follow-up period, there was no nonunion in all patients with ICBG, and a small amount of bone resorption in the cancellous area was found. Expansive callus formation was observed in all cases, which was consistent with secondary fracture healing. Healing of the host bone and the grafted iliac crest block occurred in all cases within 12 months, and the number of cortices was 3.3 ± 0.7. The fusion of cortical bone at each follow-up time was summarized in Table 3. Several postoperative complications were observed. In the early postoperative period, dorsalis pedis hypoesthesia was found in 2 patients, which was recovered within 6 months. One patient had dorsiflexion weakness of the great toe, which was completely recovered by physical therapy and functional exercise at 2 weeks after operation. Ankle joint stiffness and edema were observed in 3 patients at 6 months, which was recovered after rehabilitation training of joint function. The pain in iliac region was significantly improved in all cases within 3 months after surgery, and no herniation of abdominal contents was observed. There were no indications of injury of lateral femoral cutaneous nerve, ilioinguinal nerve and iliohypogastric nerve in all cases. Paley grading was summarized in Table 4. At the last follow-up, 3 patients experienced joint stiffness and discomfort, which were divided in Group general. 8 patients had complete recovery of joint function with no pain after rehabilitation. Mild joint stiffness occurred in 9 patients, and mobility was not affected. Joint stiffness in the later stages of treatment may be caused by lack of exercise. The excellent and good rate of bone healing and function was 85%.

**Table 1:** Participants details in the study (Sex, Age, Bone defect, Last follow-up time)

Case No	Sex	Age (years)	Bone Defect(cm)	Follow-up Time (Months)
1	F	35	10.5	30
2	F	42	12.0	25
3	M	66	11.5	25
4	F	35	13.7	33
5	M	44	17.0	24
6	F	43	15.2	24
7	F	50	14,4	26
8	F	39	15.6	28
9	M	61	14.1	28
10	M	55	10.8	30
11	F	58	11.1	28
12	F	58	13.0	30
13	M	46	14.1	24
14	M	55	16.5	26
15	F	42	15.5	28
16	M	57	15.1	26
17	F	46	14.0	24
18	M	59	11.1	29
19	M	54	14.7	30
20	M	60	11.9	30

**Table 2:** Visual analogue scale (VAS) and Oswestry disability index (ODI) score of the participants

Time Point	VAS (0-100)	ODI (0-100)
1 day	77.2 ± 4.4	84.7 ± 6.6
3 weeks	29.2 ± 2.2	74.7 ± 4.2
6 weeks	23.2 ± 2.7	68.9 ± 3.3
3 months	17.1 ± 2.0	52.5 ± 2.5
6 months	15.5 ± 2.2	32.5 ± 3.1
12 months	12.1 ± 2.9	15.6 ± 2.2
24 months	8.8 ± 1.9	11.1 ± 1.8

Oswestry Disability Index (ODI) was used to evaluated postoperative limb function

Visual analogue scale (VAS) was used to evaluated postoperative pain in iliac region

**Table 3:** Bone healing at each follow-up

Follow-up Time	3 W	6 W	3 M	6 M	12 M	24 M
Bone Healing (cortices)	0	0	0.5 ± 0.5	1.5 ± 0.5	3.3 ± 0.7	3.5 ± 0.5

Follow-up Time: W means Weeks, M means Months

Bone healing means 3 or 4 cortices fusion occurred

**Table 4:** Paley grading of participants

Group	n	Excellent	Good	General	Poor	Excellent and good rate (%)
	20	8	9	3	0	85

Paley grading was used to evaluated bone healing and function

## Discussion

A critical-sized tibial bone defect was reconstructed with a large segmental iliac structural bone graft, accompanied with locking compression plate. Satisfactory clinical and radiological results were obtained in all cases. Ilizarov technique and vascularized fibular grafting are widely used in the treatment of CSBD [18]. There are a few studies on the treatment of CSBD with ICBG.

VFG was widely used to achieve satisfactory results in a large number of cases with CSBD [19–21]. We did not choose this surgical option for the following reasons. Firstly, potential vascular injury was an important factor we need to consider, as it can directly lead to graft failure. The current methods of monitoring vascular injury are lagged and indirect, including SPECT/CT [22], angiography and other monitoring approach. Vascular injury, vasospasm, and thrombosis cannot be detected as early as possible and treated in time to reverse the failure of surgery. Graft survival was assessed by monitoring the viability of the grafted bone, hardly to directly monitor the condition of the graft in real time. Both ipsilateral and contralateral VFG can cause instability of the knee joint and ankle joint. According to existing study, the incidence rate of complications of the donor lower limb is 10.7%, including foot drop, Great toe contracture and paresthesia [4]. In addition, the success of VFG surgery depends on the microsurgical skills of the surgeons, and the lengthy learning curve of VFG makes it a challenge for the surgeons.

The use of avascular free iliac bone graft in the treatment of large tibial bone defects with good fusion is rare. Giannoudis et al. proposed the diamond theory of fracture healing in 2007 [23], which emphasizes that biological factors, including osteoblasts, bone conduction scaffolds and osteogenic inducers, play an important role in bone healing. As the most widely used bone graft source, iliac crest contains a large number of cancellous bones, osteoinductive factors and osteoprogenitor cells, greatly improving the biological osteogenic environment of the bone defect area. Osteoprogenitor cells differentiate into mature osteocytes under the induction of osteoinductive factors. Trabecular bone structure provides a scaffold for osteocytes crawling. The good biological environment of osteogenesis reduces the incidence of bone nonunion.

Bone resorption after transplantation is a common and troubling problem in clinical treatment. It has been reported that abnormal blood supply at the bone graft site [24], stress shielding effect of internal fixation [25], and active osteoclast function are potential causes of bone resorption [24]. Bone resorption usually occurs in the cancellous bone rather than in the cortex. Therefore, iliac crest, which retains cortical bone and a large amount of cancellous bone at the same time, is an ideal bone graft. Both 2 grafted iliac block contains 4 parts, including 3 cortex bone (inner plate, outer plate and iliac crest) and cancellous bone. Limited part of the cortical bone of the iliac crest was removed to increase the contact area of cancellous bone and the tibia to reduce the incidence rate of nonunion. Most of the cortical bone was retained to ensure the bone strength and low bone absorption rate. Cortical bone screws were used to integrate two iliac blocks to form a longer one to treat CSBD. After long-term follow-up, massive bone resorption was not found and the bone healing rate and lower limb function were satisfactory.

The complication rate of ICBG is 2–49% [13], including iliac spine depression, paresthesia caused by nerve injury, abnormal gait and skin scar [26]. Postoperative pain in iliac donor area is most common complication. Existing studies revealed the incidence rate of postoperative pain in donor iliac area was 13–20% [26, 27]. Surprisingly, there was no significant difference in VAS scores at 6w, 3 m, 6 m, and 12 m in patients who underwent large iliac crest bone graft, which did not increase pain experience [28]. No significant iliac donor pain was observed during follow-up. An unconscious sense of iliac bone loss was not mentioned. Ankle stiffness was observed in 3 patients, with a lower incidence compared to the ilizarov technique. Besides, there was no significant difference in healing time and weight -bearing time of tibial defect treated by ICBG compared with VFG [29] and ilizarov technology [30]. Therefore, large ICBG for the treatment of CSBD can be advocated.

The study also has its limitations. As a result of the iliac bone harvesting, the iliac crest bone defect was inevitable. Therefore, the risk of herniation of abdominal contents may be higher in patients with weak abdominal muscles than that underwent limited iliac bone grafting, although this complication was not found during follow-up. In addition, for cases with CSBD over 17 cm, our existing work and experience are limited, which is what we will further study in the future.

## Conclusion

Free large iliac crest bone grafting has provided good clinical and imaging results in critical-sized tibial bone defect cases (> 5 cm), thus confirming its acceptability as an acceptable surgical procedure.

## Data Availability

The datasets analyzed in this study are available from the corresponding author upon reasonable request.
